# Prospective study of cetuximab and gemcitabine in combination with radiation therapy: feasibility and efficacy in locally advanced pancreatic head cancer

**DOI:** 10.1186/s13014-015-0564-8

**Published:** 2015-12-15

**Authors:** Michele Fiore, Lucio Trodella, Sergio Valeri, Domenico Borzomati, Barnaba Floreno, Edy Ippolito, Pasquale Trecca, Luca Eolo Trodella, Rolando Maria D’Angelillo, Sara Ramella, Roberto Coppola

**Affiliations:** Radiotherapy Unit, Campus Bio-Medico University, Via A. del Portillo, 21, 00128 Rome, Italy; Department of General Surgery, Campus Bio-Medico University, Rome, Italy

**Keywords:** Pancreatic cancer, Radiochemotherapy, Cetuximab, Patient selection

## Abstract

**Background:**

Radio-chemotherapy is one of the steps of multidisciplinary management in locally advanced pancreatic cancer. The Epidermal Growth Factor Receptor (EGFR) plays an important role in the disease pathway. The purpose of this prospective study is to evaluate the feasibility and the efficacy of radiotherapy in combination with gemcitabine and EGFR targeting therapy for patients with locally advanced disease.

**Materials and methods:**

From November 2008 through January 2012, 34 patients were included in this study. In all cases an accurate pre-treatment staging including CT scan, Endoscopic Ultra-Sonography (EUS), 18F - fluorodeoxyglucose (18F-FDG) PET-CT and laparoscopy with peritoneal washing was performed. External beam radiation was delivered with a total dose of 50.4 Gy (1.8 Gy per fraction). Patients were treated using 3D- conformal radiotherapy, and the clinical target volume was the primary tumor and involved lymph nodes. Gemcitabine 300 mg/m^2^ and Cetuximab were given weekly during radiation therapy.

**Results:**

Ten patients (29.4 %) were excluded from the protocol because of the evidence of metastatic disease at the pre-treatment staging. Three patients refused radiochemotherapy. Twenty-one patients completed the therapy protocol. During the combined therapy grade 3–4 toxicities observed were only haematological (leukopenia 47,6 %, trombocytopenia 4.8 %, elevated gamma-GT 23.8 %, elevated alkaline phosphatase 4,8 %). Non-haematological toxicity grade 3–4 was never reported. Post-treatment workup showed partial response in five patients (24 %), stable disease in 11 patients (52 %) and disease progression in 5 patients (24 %). Two-year Local Control was 49 % (median, 18.6 months), 2-year Metastases Free Survival was 24 % (median, 10.8 months). One and two-year Overall Survival were 66 % and 28 % respectively, with a median survival time of 15.3 months.

**Conclusions:**

The combination of cetuximab and gemcitabine with concurrent radiation therapy provides a feasible and well tolerated treatment for locally advanced pancreatic cancer. Patients’ selection is crucial in order to treat patients appropriately.

## Introduction

Pancreatic Ductal Adenocarcinoma Cancer (PDAC) is the 10th most commonly diagnosed cancer and the 4th leading cause of cancer death in the U.S. The American Cancer Society estimates that 48,960 new cases and 40,560 deaths will occur in the U.S. in 2015 [1].

The only chance of cure and prolonged survival for PDAC patients (pts) is surgical resection. However, only 20 % of cases can be considered surgically resectable at diagnosis, while approximately 30 %-40 % of patients are affected by a locally advanced disease. The best therapeutic option for this large subset of patients has been the topic of the scientific debate in the last decade. A number of authors proposed neoadjuvant therapies for pancreatic cancer to better achieve tumor control and surgical radical resectability [2], as in several GI malignancies [3–5].

Concomitant radio-chemotherapy (RCT) with gemcitabine has shown an improvement of local control and overall survival in the neoadjuvant setting [6].

In recent years preclinical data suggest that addition of Epidermal Growth Factor Receptor (EGFR) inhibitors can increase the activity of the use of gemcitabine and radiation in pancreatic cancer cell lines and tumors [7, 8].

Cetuximab, a monoclonal antibody that specifically binds to the EGFR, overexpressed in pancreatic cancer, has been shown in vivo and in vitro to enhance radiosensitivity, promote radiation induced apoptosis, decrease cell proliferation, inhibit radiation-induced damage repair and inhibit tumor angiogenesis [9].

The purpose of this study is to evaluate the feasibility and the efficacy of RCT with gemcitabine in combination with cetuximab for patients with locally advanced PDAC.

## Materials and methods

The trial has been performed as a single-center one-armed phase II study.

From November 2008 through January 2012, 34 consecutive patients affected by histologically proven pancreatic cancer fulfilling the inclusion criteria listed below were included in the study.

Only patients that met the following inclusion criteria were included in the treatment protocol: proven cytological or histological diagnosis of PDAC; age between 18 and 75 years; no previous RCT; 0-I ECOG performance status; adequate cardiac, liver and kidney function and a good bone marrow reserve. Patients were included if they had borderline resectable or unresectable pancreatic tumours. Definitions of borderline and unresectable disease were as per NCCN guidelines. Borderline resectable tumors were defined by venous involvement of the superior mesenteric vein (SMV) or portal vein (PV), gastroduodenal artery encasement, or abutment of the superior mesenteric artery (SMA) up to 180°. Unresectable disease was defined by greater than 180° of SMA involvement, SMV/PV occlusion that is not amenable to reconstruction, or aortic or inferior vena cava (IVC) invasion or encasement.

The exclusion criteria were: resectable and metastatic disease; previous or concomitant malignant disease; one or more of the following clinical conditions: infection, pregnancy or breast-feeding, liver failure, kidney failure, Pa O2 < 65 mmHg, Pa CO2 > 40 mmHg, mental disability.

In all cases a thorough pre-treatment staging was performed, including:

- clinical examination (ECOG performance status);
- complete blood tests and tumor markers (CEA, CA 19–9, CA 125);
- multilayer CT scan (MS-CT) with and without contrast enhancement;
- endoscopic ultrasonography (EUS) with fine needle aspiration biopsy;
- laparoscopy (LPS) with peritoneal washing;
- PET-CT with 18F-2-fluoro-2-deoxy-D-glucose (FDG).

Jaundiced patients underwent endoscopic biliary stenting before or during RCT.

The design of this study was approved by the Institutional Review Board.

Each patient underwent CT-based planning before treatment. Each planning CT scan was obtained using a Simens 16-CT simulator (Siemens Medical System).

Radiotherapy target volumes were established by CT scan and PET-CT scan. The Clinical Target Volume (CTV) included the tumor and involved lymph nodes (>1 cm on CT scan and PET positive). The Planning Target Volume (PTV) was defined by CTV with a safety margin of 1 cm in all directions to include organ motion and set-up errors. Patients were fixed during therapy by individual immobilization devices.

External beam radiation was delivered to total dose of 50.4 Gy with fractionation of 1.8 Gy daily for 5 days a week. Patients were treated using 3D-conformal radiotherapy. Organs at risk for radiation-induced side effects were contoured on the planning CT and dose volume histograms (DVH) were calculated. Doses to the liver, kidneys and spine were not to exceed the stated dose tolerance guidelines used in the study. Maximum allowable radiation dose to the spinal cord was 40 Gy, no more than 50 % of the total liver volume was planned to receive more than 30 Gy (V30 < 50 %), and no more than 30 % and 50 % of the contralateral and ipsilateral kidney, respectively, were planned to receive more than 20 Gy (V20 < 30 % and 50 %). The minimal and maximal doses in the target volume were specified.

All treatments were delivered with a 15-MV linear accelerator (Varian Medical System) with a multifield isocentric technique using a multileaf collimator. Four axial fields were commonly placed, although non-coplanar techniques were also used. All fields were treated daily. A quality-control protocol was applied for all patients with the periodical acquisition of digital portal images to evaluate the precision of the set-up.

Systemic therapy started the same day as radiation therapy and consisted of Gemcitabine 300 mg/m^2^ given weekly and Cetuximab given as loading dose 400 mg/m^2^ on day 1, and sequential Cetuximab 250 mg/m^2^∕week simultaneously with radiation.

Patients were evaluated using a directed history and physical examination weekly during treatment.

The occurrence and nature of any adverse events were recorded according to the National Cancer Institute Common Toxicity Criteria (version 3.0) scale.

Approximately 4 weeks after the completion of RCT, restaging consisting of clinical examination, laboratory test, tumor markers, CT scan, PET-CT scan was performed.

The tumor response was defined by CT scan and PET-CT scan according to the World Health Organization.

The study protocol was approved by the independent Ethics Committee of our University.

## Results

### Patients’ selection

Between November 2008 and January 2012, 34 patients (F:18; M:16) entered in the protocol. Twenty-four (70 %) patients were diagnosed based on cytology, ten (30 %) patients had histologically-confirmed positive biopsy. The median age was 68 years (range 36–75 years). All patients showed a good performance status (ECOG Score 0) and none of them had undergone previous chemotherapy or radiotherapy. In almost all cases the tumor was located in the head of the pancreas. Resectability status was accorded to the NCCN guidelines. Eighteen patients (86 %) had locally advanced unresectable tumors, three patients (14 %) had borderline resectable disease, on the basis of the involvement of less than 180 degrees of the superior mesenteric artery or involvement of the hepatic artery within 1 cm of the celiac axis. All patients underwent a diagnostic laparoscopy to verify occult peritoneal and/or hepatic metastases and to perform peritoneal washing. Laparoscopy was positive (presence of hepatic and/or peritoneal metastases and neoplastic cells in peritoneal washing) in 8 patients (23.5 %): neoplastic cells at peritoneal washing in 2 pts, histologically proven hepatic metastases in 3 pts, peritoneal carcinomatosis in 3 pts, unrecognized on CT scan. Moreover all patients performed PET-CT with 18F-2-fluoro-2-deoxy-D-glucose (FDG) resulting negative for distant disease in 32 patients (94.1 %) and positive disease in 2 (5.9 %): one patient with pulmonary metastasis, one patient with sovraclavear metastatic lymph node.

According to the results of preoperative workup, 10 patients (29.4 %) had a metastatic disease and were therefore excluded from the protocol. Table 1 explains the results of the different staging methods for the 10 patients with metastatic disease.

**Table 1:** Results of the different staging methods for the 10 patients with metastatic disease

Patients	CT scan	PET-CT scan	Laparoscopy
1			X
2			X
3			X
4			X
5			X
6			X
7	X	X	
8			X
9		X	
10			X

After pre-treatment selection 3 patients refused RCT. Thus 21 patients were treated.

Demographic and clinical characteristics of these patients are listed in Table 2.

**Table 2:** Enrolled patients’ characteristics

Characteristic	No of patients (N = 21)	%
Age (years)		
Median	67	
Range	43–75	
Sex		
Male	8	38
Female	13	62
ECOG performance status		
0	21	100
1	0	0
CA 19–9 at diagnosis, U/mL		
Median	1028,15	
Range	0–6688	
Tumor localization		
Head	21	100
Body/Tail	0	0
Resectability status		
Borderline resectable	3	14
Unresectable	18	86

### Treatment-related toxicity

All twenty-one patients completed the therapy protocol.

During RCT the most frequent all-grade toxicities were: haematological (anemia 76,2 %, leukopenia 80,9 %, thrombocytopenia 71,4 %), nausea (47,6 %), fatigue (47,6 %) and elevated gamma-GT levels (72,6 %).

No patient showed any clinical adverse events due to infusion of cetuximab. Three patients (14.3 %) had grade 1 acneiform rash and three patients (14.3 %) grade 2. Only one patient had grade 1 onychopathy.

The most common side effects during therapy were haematological events.

G3 leukopenia and neutropenia occurred in 38,1 % and 19 % of patients respectively. Two patients (9,5 %) developed transient grade 4 leukopenia , and one patient grade 4 neutropenia not associated to fever. There were no cases of grade 3–4 anemia and grade 4 thrombocytopenia. Grade 3 trombocytopenia occurred in 4.8 % of patients.

Gastrointestinal toxicity consisted of nausea (33,3 % for grade 1 and 14,3 % for grade 2), vomiting (14,3 % for grade 1 and 4,8 % for grade 2) and diarrhoea (4,8 % for grade 1).

Fatigue and anorexia of grade 1 occurred in 38,1 % and 4,8 % of patients, and grade 2 in 9,5 % and 4,8 % of patients respectively.

No patients developed grade 3–4 elevated AST/ALT and total bilirubin. 4,8 % of patients had grade 2 of hyperbilirubinaemia and 14.3 % of patients grade 2 of hypertransaminasaemia.

Three patients (19 %) had grade 3 of elevated gamma-GT and one patient (4,8 %) grade 4. Elevated alkaline phosphatase of grade 3 occurred in one patient (4,8 %).

In ten patients with hematologic grade 3–4 toxicity the treatment was interrupted (median time of interruptions was 5 days, range 2–8 days). Granulocyte colony stimulating factors were administered in three patients (14.2 %) with G4 leukopenia/neutropenia. There were no chemoradiation-associated deaths.

The toxicity data are summarized in Table 3.

**Table 3:** Toxicity profile of chemoradiation with Cetuximab in all 21 patients

Toxicity	Grade 1		Grade 2		Grade 3		Grade 4		All grades	
	No. of events	%	No. of events	%	No. of events	%	No. of events	%	No. of events	%
Hematologic										
Anemia	10	47,6	6	28,6	0	0	0	0	16	76,2
Leukopenia	2	9,5	5	23,8	8	38,1	2	9,5	17	80,9
Granulocytopenia	5	23,8	8	38,1	4	19	1	4,8	18	85,7
Thrombocytopenia	12	57,2	2	9,5	1	4,8	0	0	15	71,4
Constitutional										
Fatigue	8	38,1	2	9,5	0	0	0	0	10	47,6
Anorexia	1	4,8	1	4,8	0	0	0	0	2	9,5
Gastrointestinal										
Nausea	7	33,3	3	14,3	0	0	0	0	10	47,6
Vomiting	3	14,3	1	4,8	0	0	0	0	4	19
Diarrhea	1	4,8	0	0	0	0	0	0	1	4,8
Liver and biliary										
Elevated total bilirubin	1	4,8	1	4,8	0	0	0	0	2	9,5
Elevated AST/ALT	6	28,6	3	14,3	0	0	0	0	9	4,3
Elevated Alkaline phosphatase	5	23,8	3	14,3	1	4,8	0	0	9	4,3
Elevated Gamma-GT	6	28,8	5	23,8	4	19	1	4,8	16	76,2
Acneiform Rash	3	14,3	3	14,3	0	0	0	0	6	28,8

### Treatment efficacy

All patients enrolled were evaluated for clinical response. The average time of restaging after radiochemotherapy was 3.6 weeks (range 2.6-5 weeks).

Post-treatment CT scan showed that 5 patients (24 %) had a partial response, in 11 patients (52 %) the disease resulted stable and 5 patients (24 %) experienced disease progression. If post-treatment PET-CT scan is considered, all patients showed a reduction in the value of SUVmax (Standard Uptake Value maximum).

Seven patients (33.3 %) underwent surgical radical resection, and one patient (14.7 %) achieved a complete pathological response after combined radiochemotherapy.

Twenty patients (95.2 %) received further sequential chemotherapy for a median of 4 months (range, 1 to 18 months). Fifteen patients received gemcitabine, four received other gemcitabine-based chemotherapy, and one received single-agent capecitabine. Eighteen (90 %) had no grade 3 or higher toxicity during chemotherapy. Two patients (10 %) experienced grade 3 gastrointestinal toxicities (eg, nausea, vomiting, and/or diarrhea) that required intravenous fluids but not hospitalization. Dose adjustments using standard dose-modification tables were based on the worst toxicity observed during the previous cycle.

The median follow-up was 23.3 months (range, 6 to 78 months).

**Fig. 1:**
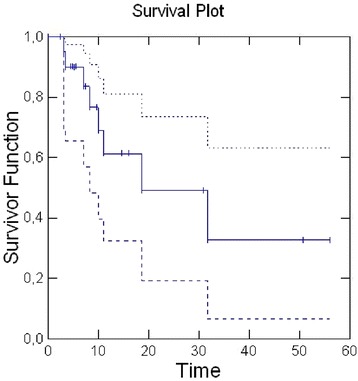
Kaplan-Meier method for Local Control. The solid line represents the curve of Local Control; the dashed lines are the upper and lower limits of Local Control curve

**Fig. 2:**
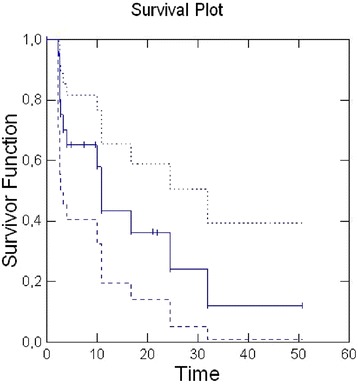
Kaplan-Meier method for Metasases Free Survival. The solid line represents the curve of Metastases Free Survival; the dashed lines are the upper and lower limits of the Metastases Free Survival curve

Two-year Local Control (LC) was 49 % (median, 18.6 months; 95 % CI: 8.2–41), 2-year Metastases Free Survival (MFS) was 24 % (median, 10.8 months; 95 % CI: 3.2-31.9) [Figs. 1, 2].

**Fig. 3:**
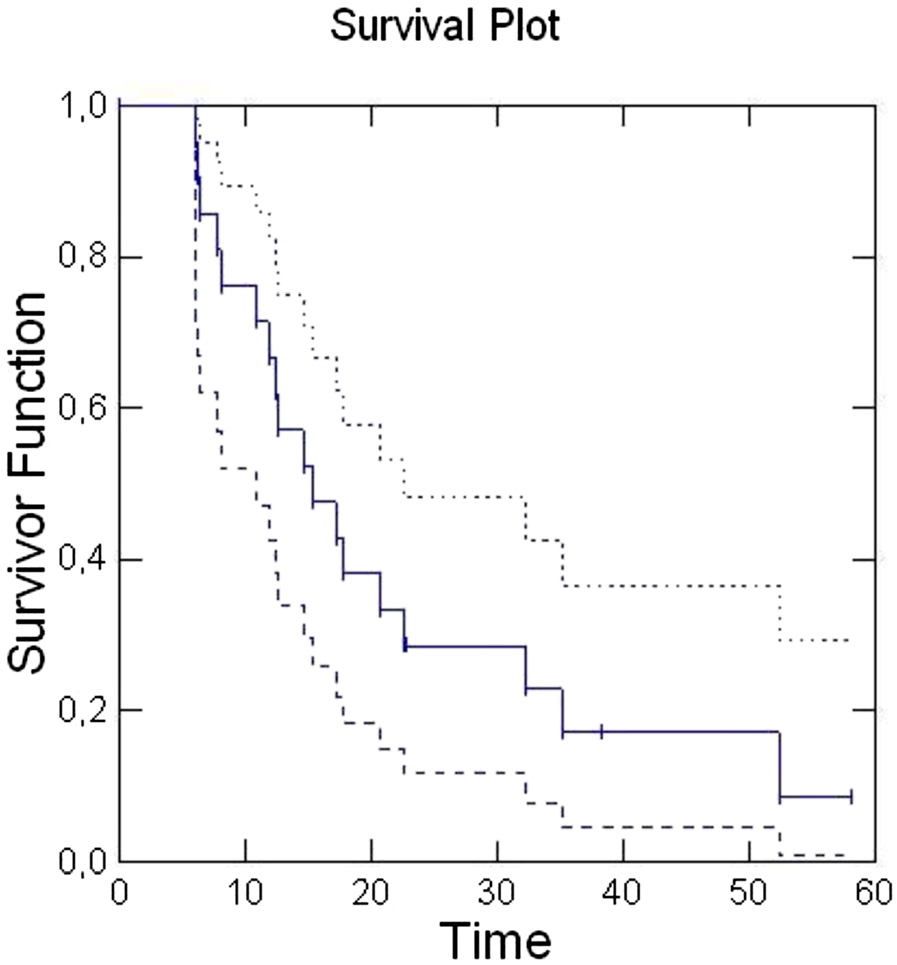
Kaplan-Meier method for Overall Survival. The solid line represents the curve of the Overall Survival; the dashed lines are the upper and lower limits of the Overall Survival curve

One-year Overall Survival (OS) and two-year OS were 66 % and 28 % respectively, with a median survival time of 15.3 months (95 % CI:10.8–22.5) [Fig. 3].

The initial site of progression was local in 3 patients, distant in 11 patients and local and at distant in two patients. Of patients who experienced local failure, three failed outside the radiotherapy fields at lomboaortic lymph nodes, two patients had ‘in-field’ recurrences. The systemic progression affected the liver in 6 patients, three patients showed peritoneal carcinomatosis (one patients experienced both liver and peritoneal progression), and two patients had lung and ovarian metastases. Four patients received palliative chemotherapy.

At the time of evaluation, three of the seven patients who underwent radical surgery are alive, two patients without evidence of disease, and one is continuing chemotherapy for distant progression.

## Discussion

The optimal treatment strategy for locally advanced PDAC is still controversial, and despite considerable progress in oncology the poor prognosis of patients has not significantly improved. Thus there is clearly a need for additional therapeutic strategies. Improved biologic understanding of the disease and investigation of novel mechanism-based therapeutics are required to facilitate the development of more effective treatments with reduced toxicity. Modern radiotherapy has important roles in controlling local disease: to prevent disease symptoms, increase the possibility of resection, and extend survival. However, when combined with chemotherapy to maximize disease control, the effectiveness of treatment is limited by the radiation doses that can be given safely, due to the risk of toxicity in surrounding radiosensitive abdominal structures. In our opinion, the selection of patients affected by this disease is a crucial issue in the debate of integrated treatments. Advances in diagnostic imaging, with the improvement in the quality of computed tomography (CT) scanning, the input of PET-CT scan and the use of laparoscopy make it possible. For our diagnostic workup protocol we have combined imaging and laparoscopy to better select patients for radiochemotherapy and to select them for surgery after the combined modality.

After preoperative staging, ten patients (29.4 %) had metastatic disease and were excluded from the protocol, while 24 patients (70.6 %) were enrolled for the combined therapy.

Our diagnostic workup, including CT scan, PET-CT scan and laparoscopy, could be a model useful to clinicians who treat pancreatic cancer.

Better patient selection and multimodality treatment are crucial concepts to improve outcomes [10, 11]. Gemcitabine (difluorodeoxycytidine), an indipendent cytotoxic agent, is a nucleoside analog with potent radiosensitizing effects [6–8]. According to the results of randomized trials gemcitabine provides improvement in survival and clinical benefit compared with 5-fluorouracil (5-FU) treatment in advanced PDAC patients [12, 13]. For this superiority as both systemic therapy and potent radiosensitizing agent [14], we realized a protocol of treatment that included the use of preoperative gemcitabine-based chemoradiation. On basis of literature data a single agent should not be considered for locally advanced PDAC patients. Indeed therapies towards multiple targets may be more beneficial.

New therapeutic approaches involve the identification of a number of molecular targets that may be responsible of the resistance of cancer cells to radiation or to other cytotoxic agents. Among these EGFR has been a molecular target of considerable interest and investigation [9].

Cetuximab is a monoclonal antibody that specifically binds to the EGFR, thereby inhibiting downstream signal transduction pathways.

Based on these considerations, we designed a clinical prospective protocol consisting of preoperative gemcitabine-based chemoradiation adding cetuximab to assess the feasibility and efficacy of this therapeutic strategy in patients with locally advanced pancreatic cancer.

Our regimen of weekly cetuximab and gemcitabine delivered with concurrent radiation therapy resulted in a good profile of tolerability in patients with pancreatic cancer.

Crane et al. [15] reported the results of a phase II trial of induction gemcitabine, oxaliplatin, and cetuximab followed by radiation (50.4 Gy) with concurrent capecitabine and cetuximab in 69 patients with locally advanced pancreatic cancer. Cetuximab (500 mg/m^2^) was started on day 1 of chemotherapy and was continued every 2 weeks during chemotherapy and chemoradiotherapy. Fifty-one patients (74 %) had unresectable tumors; 16 patients (23 %) had borderline resectable tumors due to vascular abutment, whereas 2 patients (3 %) had borderline resectable disease only on the basis of advanced regional adenopathy. Median follow-up was 16.3 months for all patients and 20.9 months for living patients; median progression-free survival (PFS) and OS were 12.5 and 19.2 months, respectively. In this study, six patients (8.7 %) had infusion reactions to cetuximab, and two of these were grade 3. Acneiform rash was 54 % and 3 % for grades 2 and 3, respectively; it correlated with improved survival. In our study only three patients (14.3 %) had grade 2 acneiform rash, no patient showed grade 3 toxicity. There was no correlation between acneiform rash and clinical outcomes.

Our results agree with those of a phase I study evaluating cetuximab, gemcitabine and radiotherapy for locally advanced pancreatic cancer [16]. Recent results published by Arnoletti et al. have reported that the dose escalation was performed with gemcitabine (0–300 mg/m^2^) and cetuximab (400 mg/m^2^ loading dose and 250 mg/m^2^ weekly). The results of this study showed that 96 % of patients experienced grade 1–2 adverse events and 9 % had grade 3–4 adverse events. As in our study, there were not serious treatment-related events, and no patients deaths were associated to drug toxicity and to radiation schedule.

In two phase I studies reported both in abstract forms, the scheme of gemcitabine (300 mg/m^2^) and cetuximab (400 mg/m^2^ loading dose and 250 mg/m^2^∕week) with concurrent radiation therapy was tolerated and appeared to be effective [17, 18].

Our results differ widely from those of another recently published phase I study, in which patients with locally unresectable pancreatic cancer were treated with gemcitabine (200 mg/m^2^ before dose escalation) plus cetuximab (400 mg/m^2^ loading dose followed by 250 mg/m^2^∕week) concurrent with radiation therapy (planned dose 50.4 Gy) [19] . Of the nine patients enrolled in this study, one was withdrawn before receiving the initial dose of cetuximab because of a decline in performance status; the remaining eight patients in which the tolerance to treatment was assessed presented a various baseline performance status. Five patients received more than the loading dose of cetuximab, and only one patient completed all doses of chemotherapy and radiotherapy.

The authors found a significant toxicity grade, including a high rate of infusion reactions, and they concluded that this combination should be approached with caution. This severe toxicity observed could be related to the traditional radiotherapy treatment volumes, which included primary tumor and prophylactic drainage nodes in the definition of clinical target volumes.

In our study, on the contrary, all patients completed the therapy protocol. We did not experience severe adverse events, included infusion reactions. The prophylactic use of chlorphenamine is likely to have prevented them.

The use of PET for target volume definition and delineation in GI tract tumors is only recently being investigated. In pancreatic cancer the interest is rapidly growing. Topkan et al. have compared CT- and PET/CT-based target volume delineation and the effects of these different modalities on 3D-conformal radiotherapy planning and radiation doses to critical organs. The authors demonstrated that PET-CT-based target volume contouring significantly increases the GTV (Gross Tumor Volume) and the PTV compared to CT-based contouring without increasing tissue toxicity in a clinically meaningful way. The authors utilized PET data and included the regional lymph nodes which appeared to be metabolically involved on PET scan but not on CT [20]. As this study suggests, the largest potential benefit of incorporating PET into RT planning for locally advanced pancreatic cancer may be the reduction in geographic misses associated with CT-based planning and the potential reduction in loco-regional treatment failure. On the basis of these considerations, we contoured small treatment volumes, including the pancreatic tumor and the involved lymph nodes. Radiation treatment volumes were defined not only based on the CT scan, as shown by the evidence in the literature, but also by using biological information obtained through PET-CT scan. In our cohort median PTV volume was 267.7 cc (range 130.6-797.6 cc). No patient developed grade 3–4 gastrointestinal toxicity. This may have been aided by limited-field radiotherapy.

In our study, RCT treatment, conducted on small volumes (only the areas biologically active) was safe and well tolerated with a manageable toxicity. None of 21 patients developed intestinal bleeding, a complication almost constant, variable from 3 % to 20 % [12, 13, 21–27]. At the same time the reduction of radiotherapy treatment volumes did not compromise the outcomes of survival and locoregional control. The median survival was 15.3 months, resulting improved in comparison with other studies of the literature [12, 13, 21–27]. Crane et al. retrospectively compared the efficacy of concurrent gemcitabine-based RCT with that of concurrent 5-FU-based RCT in patients with unresectable pancreatic cancer. Patients treated with gemcitabine had a median survival time of 11 months compared to 9 months of patients treated with 5-FU [12]. Brunner et al. published a retrospective analysis of patients treated with radiotherapy and 5-FU- or gemcitabine-based chemotherapy followed by additional chemotherapy with gemcitabine. In these patients the median survival time was 13 months [13]. In a prospective randomized study Li et al. investigated the efficacy of gemcitabine-concurrent RCT vs 5-FU based-RCT for locally advanced pancreatic cancer. The median survival was 14.5 months for gemcitabine-based RCT patients compared with 6.7 months for 5-FU-based RCT patients. The 1- and 2-year survival rate was 56 % and 15 % for gemcitabine-RCT compared with 31 % and 0 % for 5-FU-RCT, respectively [21]. In Huguet et al. trial, patients were treated with definitive gemcitabine-based chemoradiation if no progression was noted after initial induction chemotherapy. The median survival was 15 months [22]. In McGinn et al. study, weekly full-dose gemcitabine was administered concomitantly to involved-field irradiation, with a median survival time of 11.6 months [23]. In Muler et al. study [24] and in Kawakami et al. cohort [25] the median survival times were 12.9 months and 7.1 months, respectively. Goldstein et al. assessed the efficacy of a specific three-dimensional conformal radiotherapy technique with concurrent continuous infusion of 5-FU sandwiched between gemcitabine chemotherapy in patients with locally advanced pancreatic cancer. The median survival time in this study was 11.7 months, with the 1-year survival rate of 46.3 % [26]. Moreover, the results of SCALOP trial showed that the median overall survival was 15.2 months in the capecitabine-based RCT group and 13.4 in the gemcitabine-based RCT group [27].

Our study demonstrated distant relapses as dominant treatment failure, emphasizing the need to find more effective systemic agents. The improvement of local control and, possibly, disease downstaging allowing surgical resection are important endpoints. The question remains how to improve local control without worsening the tolerability of the treatments. The use of modern radiation therapy procedures, such as contrast enhancement four-dimensional computed tomography (ce-4DCT), the radiation dose escalation of the primary tumor with intensity modulated radiation therapy (IMRT) and stereotactic body radiation therapy (SBRT) and better target selection could help to solve this problem.

## Conclusions

Our prospective study gives us the opportunity to emphasize, first of all, that in the treatment of pancreatic cancer, more than in others, the selection of patients, using the most modern imaging techniques and laparoscopy, is a crucial issue in order to treat these patients in the most appropriate way and to make appropriate assessments on patients’ compliance and response to therapies. To our knowledge, this is the first study in which this modality of patient selection is designed, allowing to exclude from locoregional treatment patients with early and rapidly progressive pancreatic cancer disease. Our diagnostic workup, including CT scan, PET-CT scan and laparoscopy, could be a model useful to clinicians who treat pancreatic cancer to improve patients' selection. Moreover, the combination of gemcitabine, cetuximab with concurrent radiation therapy, with smaller treatment volumes, is feasible and well tolerated, with encouraging results, that will allow to draw further studies combining systemic chemotherapy to radiotherapy dose intensified, in order to improve clinical outcomes.

## Abbreviations

4DCT: four-dimensional Computed Tomography
5-FU: 5-fluorouracil
CTV: Clinical Target Volume
EGFR: Epidermal Growth Factor Receptor
EUS: Endoscopic ultrasound
GTV: Gross Tumor Volume
IMRT: Intensity Modulated Radiation Therapy
LC: Local Control
LPS: laparoscopy
MFS: Metastases Free Survival
OS: Overall Survival
PDAC: Pancreatic Ductal Adenocarcinoma Cancer
PFS: Progression Free Survival
PTV: Planning Target Volume
RCT: Radio-Chemotherapy
SUV: Standard Uptake Value
SBRT: Stereotactic Body Radiation Therapy

## References

[1] Siegel RL, Miller KD, Jemal A (2015). Cancer Statistics, 2015. CA Cancer J Clin.

[2] Kleeff J, Friess H, Buchler MW (2007). Neoadjuvant therapy for pancreatic cancer. Br J Surg.

[3] Sauer R, Becker H, Hohenberger W, Rodel C, Wittekind C, Fietkau R (2004). German Rectal Cancer Study Group. Preoperative versus postoperative chemoradiotherapy for rectal cancer. N Engl J Med.

[4] Mezhirr JJ, Tang LH, Coit DG (2010). Neoadjuvant therapy for locally advanced gastric cancer. J Surg Oncol.

[5] Campbell NP, Villaflor VM (2010). Neoadjuvant treatment of esophageal cancer. World J Gastroenterol.

[6] Loehrer PJ, Feng Y, Cardenes H, Wagner L, Brell JM, Cella D (2011). Gemcitabine alone versus gemcitabine plus radiotherapy in patients with locally advanced pancreatic cancer: An Eastern Cooperative Oncology Group Trial. J Clin Oncol.

[7] Buchsbaum DJ, Bonner JA, Grizzle WE, Stackhouse MA, Carpenter M, Hicklin DJ (2002). Treatment of pancreatic cancer xenografts with Erbitux (IMC-C225) anti-EGFR antibody, gemcitabine, and radiation. Int J Radiat Oncol Biol Phys.

[8] Morgan MA, Parsels LA, Kollar LE, Normolle DP, Maybaum J, Lawrence TS (2008). The combination of epidermal growth factor receptor inhibitors with gemcitabine and radiation in pancreatic cancer. Clin Cancer Res.

[9] Krempien R, Muenter MW, Huber PE, Nill S, Friess H, Timke C (2005). Randomized phase II – study evaluating EGFR targeting therapy with Cetuximab in combination with radiotherapy and chemotherapy for patients with locally advanced pancreatic cancer – PARC: study protocol [ISRCTN56652283]. BMC Cancer.

[10] Lawrence TS, Chang EY, Hahn TM, Hertel LW, Shewach DS (1996). Radiosensitization of pancreatic cancer cells by 2,2-difluoro-2-deoxycytidine. Int J Radiat Oncol Biol Phys.

[11] Lawrence TS, Eisbruch A, Shewach DS (1997). Gemcitabine-mediated radiosensitization. Semin Oncol.

[12] Crane CH, Abbruzzese JL, Evans DB, Wolff RA, Ballo MT, Delclos M (2002). Is the therapeutic index better with gemcitabine-based chemoradiation than with 5–fluorouracil-based chemoradiation in locally advanced pancreatic cancer?. Int J Radiat Oncol Biol Phys.

[13] Brunner TB, Tinkl D, Grabenbauer GG, Meyer T, Brueckl WM, Sauer R (2006). Maintenance chemotherapy after chemoradiation improves survival of patients with locally advanced pancreatic carcinoma: a retrospective analysis of prospectively recruited patients. Strahlenther Onkol.

[14] Morgan MA, Parsels LA, Maybaum J, Lawrence TS (2008). Improving gemcitabine-mediated radiosensitization using molecularly targeted therapy: A review. Clin Cancer Res.

[15] Crane CH, Varadhachary GR, Yordy JS, Staerkel GA, Javle MM, Safran H (2011). Phase II trial of cetuximab, gemcitabine, and oxaliplatin followed by chemoradiation with cetuximab for locally advanced (T4) pancreatic adenocarcinoma: correlation of Smad4 (Dpc4) immunostaining with pattern of disease progression. J Clin Oncol.

[16] Arnoletti JP, Frolov A, Eloubeidi M, Keene K, Posey J, Wood T (2011). A phase I study evaluating the role of the anti-epidermal growth factor receptor (EGFR) antibody cetuximab as a radiosensitizer with chemoradiation for locally advanced pancreatic cancer. Cancer Chemother Pharmacol.

[17] Demols A, Mahin C, Maréchal R, Delaunoit T, Borbath I, Hendlisz A (2008). Cetuximab plus chemoradiation combined therapy for locally advanced inoperable pancreatic adenocarcima: A phase I study. J Clin Oncol Am Soc Clin Oncol Meeting Proc.

[18] Munter M, Timke C, Abdollahi A, Friess H, Jaeger D, Heeger S (2008). Final results of a phase II trial [PARC-Study ISRCTN56652283] for patients with primary inoperable locally advanced pancreatic cancer combining intensity modulated radiotherapy (IMRT) with cetuximab and gemcitabine. J Clin Oncol Am Soc Clin Oncol Meeting Proc.

[19] Chakravarthy AB, Tsai CJ, O'Brien N, Lockhart AC, Chan E, Parikh A (2012). A phase I study of cetuximab in combination with gemcitabine and radiation for locally advanced pancreatic cancer. Gastrointest Cancer Res.

[20] Topkan E, Yavuz AA, Aydin M, Onal C, Yapar F, Yavuz MN (2008). Comparison of CT and PET-CT based planning of radiation therapy in locally advanced pancreatic carcinoma. J Exp Clin Cancer Res.

[21] Li CP, Chao Y, Chi KH, Chan WK, Teng HC, Lee RC (2003). Concurrent chemoradiotherapy treatment of locally advanced pancreatic cancer: gemcitabine versus 5-fluorouracil, a randomized controlled study. Int J Radiat Oncol Biol Phys.

[22] Huguet F, André T, Hammel P, Artru P, Balosso J, Selle F (2007). Impact of chemoradiotherapy after disease control with chemotherapy in locally advanced pancreatic adenocarcinoma in GERCOR Phase II and III studies. J Clin Oncol.

[23] McGinn CJ, Zalupski MM, Shureiqi I, Robertson JM, Eckhauser FE, Smith DC (2001). Phase I trial of radiation dose escalation with concurrent weekly full-dose gemcitabine in patients with advanced pancreatic cancer. J Clin Oncol.

[24] Muler JH, McGinn CJ, Normolle D, Lawrence T, Brown D, Hejna G (2004). Phase I trial using a time-to-event continual reassessment strategy for dose escalation of cisplatin combined with gemcitabine and radiation therapy in pancreatic cancer. J Clin Oncol.

[25] Kawakami H, Uno T, Isobe K, Ueno N, Aruga T, Sudo K (2005). Toxicities and effects of involved-field irradiation with concurrent cisplatin for unresectable carcinoma of the pancreas. Int J Radiat Oncol Biol Phys.

[26] Goldstein D, Van Hazel G, Walpole E, Underhill C, Kotasek D, Michael M (2007). Gemcitabine with a specific conformal 3D 5FU radiochemotherapy technique is safe and effective in the definitive management of locally advanced pancreatic cancer. Br J Cancer.

[27] Mukherjee S, Hurt CN, Bridgewater J, Falk S, Cummins S, Wasan H (2013). Gemcitabine-based or capecitabine-based chemoradiotherapy for locally advanced pancreatic cancer (SCALOP): a multicentre, randomised, phase 2 trial. Lancet Oncol.

